# Deep learning based joint fusion approach to exploit anatomical and functional brain information in autism spectrum disorders

**DOI:** 10.1186/s40708-023-00217-4

**Published:** 2024-01-09

**Authors:** Sara Saponaro, Francesca Lizzi, Giacomo Serra, Francesca Mainas, Piernicola Oliva, Alessia Giuliano, Sara Calderoni, Alessandra Retico

**Affiliations:** 1 Medical Physics School, University of Pisa, Pisa, Italy; 2 National Institute for Nuclear Physics (INFN), Pisa Division, Pisa, Italy; 3 Department of Physics, University of Cagliari, Cagliari, Italy; 4 INFN, Cagliari Division, Cagliari, Italy; 5 Department of Computer Science, University of Pisa, Pisa, Italy; 6 Department of Chemical, Physical, Mathematical and Natural Sciences, University of Sassari, Sassari, Italy; 7 Unit of Medical Physics, Pisa University Hospital “Azienda Ospedaliero-Universitaria Pisana”, Pisa, Italy; 8 Developmental Psychiatry Unit - IRCCS Stella Maris Foundation, Pisa, Italy; 9 Department of Clinical and Experimental Medicine, University of Pisa, Pisa, Italy

**Keywords:** Multi-modal machine learning, Deep Learning, Autism spectrum disorders, ABIDE, Structural MRI, Functional connectivity

## Abstract

**Background:**

The integration of the information encoded in multiparametric MRI images can enhance the performance of machine-learning classifiers. In this study, we investigate whether the combination of structural and functional MRI might improve the performances of a deep learning (DL) model trained to discriminate subjects with Autism Spectrum Disorders (ASD) with respect to typically developing controls (TD).

**Material and methods:**

We analyzed both structural and functional MRI brain scans publicly available within the ABIDE I and II data collections. We considered 1383 male subjects with age between 5 and 40 years, including 680 subjects with ASD and 703 TD from 35 different acquisition sites. We extracted morphometric and functional brain features from MRI scans with the Freesurfer and the CPAC analysis packages, respectively. Then, due to the multisite nature of the dataset, we implemented a data harmonization protocol. The ASD vs. TD classification was carried out with a multiple-input DL model, consisting in a neural network which generates a fixed-length feature representation of the data of each modality (FR-NN), and a Dense Neural Network for classification (C-NN). Specifically, we implemented a *joint fusion* approach to multiple source data integration. The main advantage of the latter is that the loss is propagated back to the FR-NN during the training, thus creating informative feature representations for each data modality. Then, a C-NN, with a number of layers and neurons per layer to be optimized during the model training, performs the ASD-TD discrimination. The performance was evaluated by computing the Area under the Receiver Operating Characteristic curve within a nested 10-fold cross-validation. The brain features that drive the DL classification were identified by the SHAP explainability framework.

**Results:**

The AUC values of 0.66±0.05 and of 0.76±0.04 were obtained in the ASD vs. TD discrimination when only structural or functional features are considered, respectively. The *joint fusion* approach led to an AUC of 0.78±0.04. The set of structural and functional connectivity features identified as the most important for the two-class discrimination supports the idea that brain changes tend to occur in individuals with ASD in regions belonging to the Default Mode Network and to the Social Brain.

**Conclusions:**

Our results demonstrate that the multimodal *joint fusion* approach outperforms the classification results obtained with data acquired by a single MRI modality as it efficiently exploits the complementarity of structural and functional brain information.

**Supplementary Information:**

The online version contains supplementary material available at 10.1186/s40708-023-00217-4.

## Introduction

Autism spectrum disorders (ASD) are a heterogeneous group of neurodevelopmental disorders characterized by persistent deficits in reciprocal social interaction, communication, and the presence of restricted, repetitive behaviors and interests, which can include sensory processing difficulties [7]. Prevalence data from the most recent investigation conducted by the American Centers for Disease Control and Prevention reported that ASD occurs in about one in every 36 children aged 8 years in U.S. [53].

ASD is currently diagnosed through a multidisciplinary and comprehensive direct evaluation of the individual with suspected ASD, associated with gold-standard behavioral observation [51] and interview [71] performed by clinicians expert in neurodevelopmental disorders [46]. However, methods based on observation of the patient and/or interview with the parents are subjective. Therefore, neuroimaging plays a key role in identifying the neural correlates of this condition. Machine learning (ML) and deep learning (DL) techniques are gaining considerable importance in supporting the diagnosis of ASD on the basis of magnetic resonance imaging (MRI) [44, 57], though these modalities do not have yet a clinical application. The aggregation of large data collections from multiple centers is often used to overcome the problems of appropriate ML training, related to the typical limited size of datasets in this field.

For ASD research, the Autism Brain Imaging Data Exchange (ABIDE) dataset is a public neuroimaging collection that is well characterized at the phenotypic level. Two world-wide multi-site and large-scale collections were released so far, ABIDE I [22] and ABIDE II [23], jointly consisting in more than a thousand cases and as many controls. In spite of the greater sample sizes, analyses based on the ABIDE collections report highly variable classification performances [77]. Moreover, it was pointed out that multi-center MRI data suffer from significant confounding due to batch-related technical variation, called batch effects [26]. In effect, MRI acquisitions made with different scanners and/or with dissimilar acquisition protocols encode confounding information in data which, if not accounted for, may obscure the tiny differences between controls with typical development (TD) and ASD subjects [25].

Several techniques can be employed to mitigate batch effects. During the study design phase, efforts can be made to mitigate batch effects by restricting data collection to a single scanner, manufacturer, field strength, acquisition protocol, or a combination of these criteria. However, this approach may limit the capacity to collect large datasets. Furthermore, even when acquisition conditions and scanner manufacturers are carefully checked, residual differences (resulting from factors such as hardware imperfections, site or operator characteristics, or software or hardware updates) can still introduce batch effects. In the image pre-processing stage, standardizing images through techniques such as gradient distortion correction, bias field correction, and intensity normalization can help to remove batch effects. However, it is important to note that these normalization methods primarily target inter-subject variability. Consequently, they can only mitigate batch effects that overlap with inter-subject variability. In the last few years, a number of advanced strategies, which employ statistical or mathematical concepts, were developed with the aim of removing the batch effect in neuroimaging studies [36]. Fortin et al. developed a harmonization algorithm [29], as an adaptation of the ComBat method developed by Johnson et al. [40] to remove batch effects in genomics data. Even if DL approach has been recently applied on ABIDE dataset [61], at the moment, ComBat appears to be the most used harmonization approach in the field of ASD research to reduce effect size in ABIDE data collections [28, 38, 68, 80]. However, Pomponio et al. [64] have recently presented a modified version of the harmonization protocol, the *NeuroHarmonize* tool, which is suitable to harmonize pooled dataset in the presence of non-linear age trends. In a recent study published by our group [72], we demonstrated that the implementation of *NeuroHarmonize* preprocessing [64] in a multi-center analysis conducted on structural MRI (sMRI) data from ABIDE I and II collections results in a significant increase in the ASD vs. TD discrimination performance of ML classifiers.

Multi-modal machine learning is a subfield of ML that aims to develop and train models that can exploit different types of data and combine them in order to improve prediction performance [2]. Indeed, combining data from multiple modalities allows to extract more comprehensive and complementary information, resulting in better performing models compared to using a single data modality [37, 81, 83]. In particular, the *joint fusion* approach employs a neural network model to extract feature representations from each modality, which are then combined and used as inputs to another model. The fusion model’s prediction loss is back propagated to the feature extracting models to enhance the learned feature representations.

In the research field of neurogenerative disorders, fusion strategies were frequently employed in the diagnosis and prediction of Alzheimer’s disease. Neither imaging or clinical data alone are sufficient for accurately diagnosing Alzheimer’s disease in clinical practice. However, leveraging DL fusion techniques has consistently shown improvements in diagnostic performance [65, 75, 78]. Some studies reported good results in applying DL models using functional and structural MRI images demonstrating that a DL framework for multi-modality data fusion outperforms single-modality DL [1, 5, 67]. In this work, we developed a multi-modal *joint fusion* DL model, which combines structural and functional MRI to distinguish between ASD and TD subjects. Although DL models are highly efficient and accurate, their complexity makes the rationale behind their decisions unclear, thus limiting their use in clinical applications such as disease diagnosis. To address this issue in recent years, different algorithms were proposed to explain which features contribute the most to the classification results. In this work we implemented the SHapley Additive exPlanations (SHAP) [52], which utilizes optimal Shapley values derived from game theory, in order to pointing out the most important feature involved in the identification of ASD subjects.

## Materials and methods

### Participants and data description

We analyzed the T1-weighted sMRI and resting-state fMRI (rs-fMRI) data of the ABIDE I [22] and ABIDE II [23] publicly available collections. Since $97\%$ of the subjects were under the age of 40 years, we limited our study to subjects aged 5 to 40 years only, similarly to other studies in the field [33, 42]. Moreover, we restricted our analysis to male subjects, due to both the limited representation of female subjects in the ABIDE collection (less than 20% of subjects, spread over different sites and a wide age-range), and the sex differences in functional brain connectivity, characterized by predominant underconnectivity in ASD males as compared to TD males and extensive overconnectivity in ASD females as compared to TD females [6]. Since our goal is to propose a classification strategy dealing with both structural and functional information, we excluded subjects with missing multimodal MRI data after using the preprocessing pipelines. Thus, we obtained a final sample of 1383 subjects (680 ASD and 703 TD) from 35 sites. A summary of the sample sizes of the ABIDE I and II cohorts included in this study and of the participants’ average age is reported in Table 1. To allow the reproducibility of the analysis, the identification numbers (IDs) of the participants selected in the final sample are reported in Additional file 1.

**Table 1:** Number of subjects of the ABIDE I and II cohorts considered in this study. Only male subjects in the age range of [5–40] years (y) are considered.

Centers	N		Average age (y)		STD age (y)	
	ASD	TD	ASD	TD	ASD	TD
BNI_A	14	11	22.1	22.5	5.6	6.3
CALTECH	12	11	24.5	24.6	6.9	6.8
CMU	7	9	25.8	27.1	4.4	6.5
EMC_A	21	22	8.2	8.3	1.2	1.0
ETH_A	10	22	20.4	23.8	3.9	4.5
GU_A	37	26	11.0	10.8	1.5	1.6
IP_A	13	8	15.5	23.3	5.2	7.1
IU_A	8	9	20.7	24.2	3.7	5.0
KKI	13	18	10.2	10.4	1.3	1.3
KKI_A	30	62	10.4	10.4	1.6	1.3
LEUVEN_A	9	11	22.6	22.5	4.7	2.6
LEUVEN_B	10	13	13.7	14.5	1.4	1.7
MAX_MUN	15	26	20.5	23.3	9.5	7.8
NYU	62	73	14.0	16.0	6.5	6.3
NYU_A	41	27	9.5	9.2	4.6	1.8
OHSU	13	15	11.7	10.1	2.2	1.1
OHSU_A	29	27	12.1	10.3	2.2	1.7
OILH_B	7	7	21.3	23.8	2.9	4.1
OLIN	16	13	16.1	16.8	3.1	4.0
PITT	26	21	19.9	20.0	7.3	6.9
SBL	10	12	29.8	33	3.5	6.3
SDSU	10	13	14.4	14.5	1.7	1.4
SDSU_A	23	22	12.6	13.5	3.1	3.2
STANFORD	15	14	10.1	10.3	1.6	1.7
TCD_A	19	15	14.4	14.8	3.1	3.1
TRINITY	24	25	17.3	17.1	3.6	3.8
UCD_A	12	8	14.6	14.8	1.9	2.0
UCLA_A	33	26	13.2	13.4	2.3	2.3
UCLA_B	10	9	12.5	12.0	1.6	1.2
UM_A	33	33	12.4	13.6	2.3	3.3
UM_B	12	18	14.7	17.1	1.5	4.2
USM	46	36	21.0	20.9	5.7	8.1
USM_A	14	12	16.0	23.1	3.8	8.5
U_MIA_A	7	9	10.8	9.9	2.2	2.0
YALE	19	20	12.5	12.3	3.1	2.8
Total	703	680	15.7	14.9	7.0	6.2

The number of participants is provided per site and per diagnostic group, together with the average age and standard deviation of each group. *Abbreviation*: STD - standard deviation

### Image processing and feature extraction

#### Structural MRI scan

As in our previous work [72], the sMRI scans were processed with Freesurfer [27] version 6.0 with the *recon-all* pipeline.^1^This procedure includes cortical surface modelling, spherical coordinate transformation, non-linear curvature registration, automated volumetric segmentation and cortical reconstruction. Among the outputs generated by the Freesurfer processing pipeline, the following brain features were selected: the global measures and the subcortical features available in the file *aseg.stats* and the cortical features available in the bilateral files *aparc.stats*. In this way, a total number of 221 brain morphometric features were obtained. These brain descriptive characteristics can be grouped into:^2^

- 9 global quantities: left (L) and right (R) mean thickness, L and R cortex volumes, L and R cerebral white matter volume, cerebrospinal fluid volume, total gray volumes and the volume of segmented brain without ventricles;
- 26 volumes of sub-cortical structures and corpus callosum;
- 186 measures, including the volume, the mean and standard deviation of the thickness of 62 structures (31 per hemisphere) from the Desikan-Killiany-Tourville Atlas [45]: 14 in the temporal lobe, 20 in the frontal lobe, 10 in the parietal lobe, 8 in the occipital lobe and 10 in the cingulate cortex.

#### Resting-state fMRI scan

The rs-fMRI scans selected from ABIDE I and ABIDE II cohorts were processed with the Configurable Pipeline for the Analysis of Connectomes (C-PAC) [20], that includes motion correction, slice timing correction, band-pass filtering, spatial smoothing and registration. The Harvard-Oxford (HO) atlas was used to extract time series from brain regions, obtaining 110 timeseries for each subject. Seven regions were eliminated because they were not associated with any time series in a significant number of patients. In order to maximize the population of the dataset, the region was removed instead of discarding the patient’s exam. Thus, we obtained 103 timeseries for each subject. The Pearson correlation was calculated between the timeseries of pairs of regions to derive a functional connectivity (FC) matrix. The correlation values were normalized according to Fisher transformation [16] in order to make them approximately normally-distributed. Moreover, the correlation values were multiplied by $\sqrt{(N-3)}$ (where *N* is the number of timepoints) to have a unitary standard deviation. From the symmetric FC matrix, we used $N(N - 1)/2$ non-redundant values as features, obtaining 5253 connectivity features for each subject.

### Harmonization

Due to the multisite nature of the dataset, we separately harmonized the Freesurfer structural features and the functional connectivity measures using the publicly available Python package *NeuroHarmonize*,^3^ which is the state-of-the-art tool for multi-site neuroimaging analysis developed by Pomponio et al. [64]. We estimated the NeuroHarmonize model parameters on the entire cohort of control subjects, by specifying the age as a covariate, whose effect is to be preserved during the harmonization process. Finally, we applied the estimated model on the entire sample of subjects with ASD and TD controls.

### Neural network architecture: a *joint fusion* approach

We developed a multi-modal DL classification model that integrates both structural and functional information. The fusion of different data modalities can be performed at different stages of the classification process. There are three main fusion strategies: *early fusion*, *joint fusion*, and *late fusion*, as discussed in Huang et al. [37] and Acosta et al. [2]. Early fusion is the simplest approach where input modalities or features are concatenated before training a single model. Instead, the joint fusion is a more advanced technique that combines and co-learns representations of different modalities during the training process. In contrast, late fusion involves training separate models for each modality and then combining their output probabilities.

**Fig. 1:**
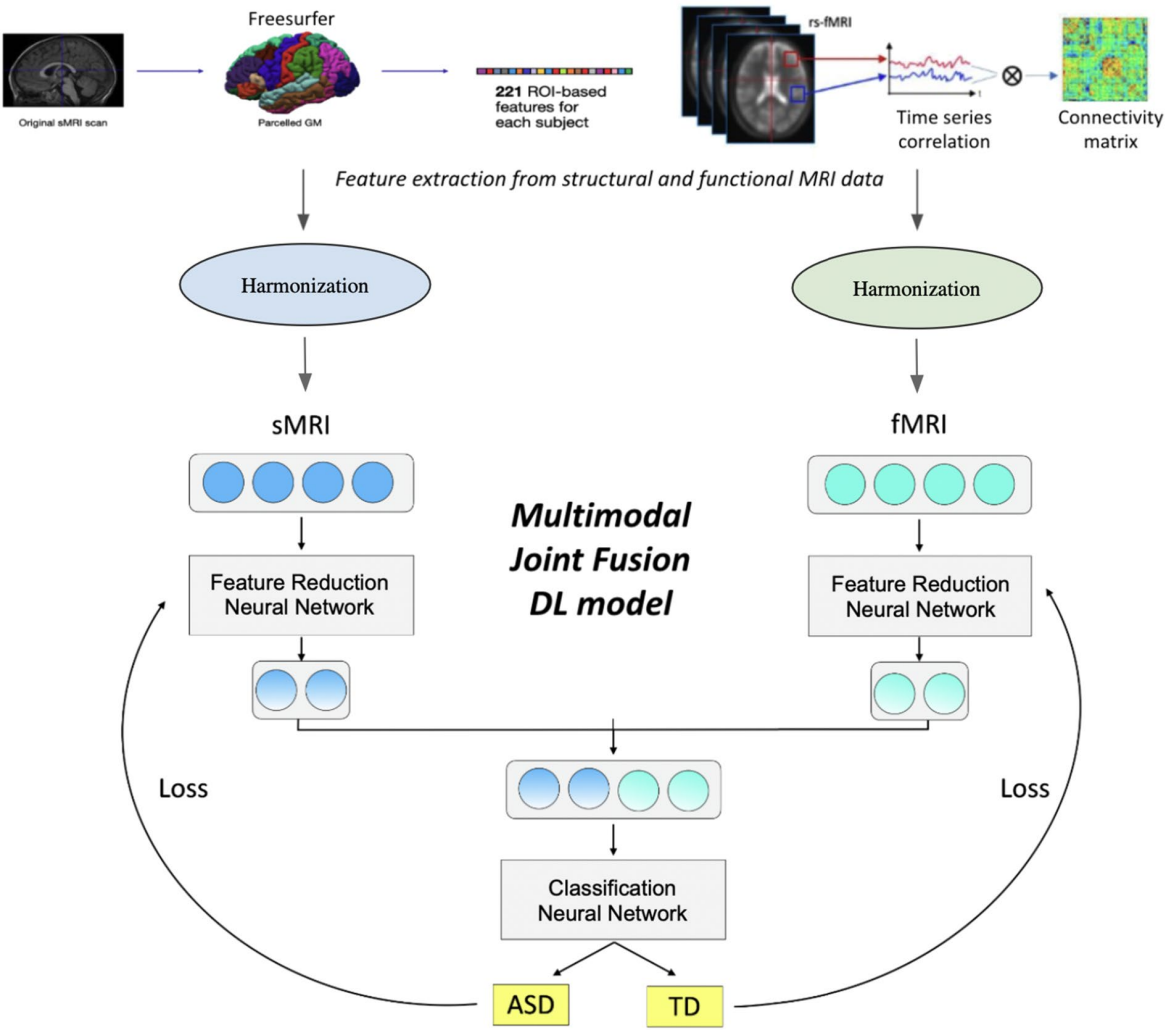
Multimodal DL model with *joint fusion* approach. The model contains a feature-reduction neural network (FR-NN) and a classification neural network (C-NN). The main advantage of this strategy is that the loss is propagated back to the FR-NN during the training (black arrows). The solid blue and cyan circles represent a starting feature set, while the shaded circles represent the fixed-length feature vectors extracted from all modalities

In this study, the ASD vs. TD classification was carried out with a DL model, consisting in a feature dimensionality reduction neural network (FR-NN) which generates a fixed-length feature representation of the data for each modality. The two vectors are then merged and passed through a classification neural network (C-NN). Specifically, we implemented the *joint fusion* approach [37], whose main advantage is that the loss is propagated back to the FR-NN during the training, thus creating informative feature representations for each data modality. The C-NN, with several layers and neurons per layer, is optimized during the model training and performs the ASD-TD discrimination. In Fig. 1 a simplified scheme of our model was shown. Moreover, in order to evaluate the improvement of using a multimodal *joint fusion* model, we also implemented models based on single data modality. Only structural or connectivity features were considered using similar NN to perform the classification. We implemented the DL model using Keras [18], a Python DL API that uses Tensorflow as backend. The model was trained using Stochastic Gradient Descent (SGD) optimizer with a learning rate of 0.001 and a momentum of 0.9 and a ReLU as the activation function. During training we minimised the binary cross entropy between the model’s predictions and the true labels for ASD and TD subjects. The model was trained for 150 epochs, moreover standard DL techniques were adopted to reduce overfitting. In particular, we used:

- batch normalisation [39], a technique which normalises the outputs of each layer for each batch of data, thus accelerating the rate of training and acting as a regularizer, reducing the internal covariate shift;
- dropout [76] which works by randomly dropping units and their connections during training. Dropout was set to 0.5 and 0.2;
- L1 regularisation which adds a penalty to the loss function and, hence, it shrinks the less important features’ coefficients, allowing for a better feature selection. The L1 regularisation hyperparameter was 0.01.

We implemented a feature scaling function (the *Scikit-learn* *RobustScaler*), that consists in the subtraction of the median and the scaling with respect to the interquartile range (IQR). The model has been trained according to a nested $10-$fold cross-validation scheme preserving the matching proportions of diagnosis (ASD/TD). The training of the model was performed in the inner CV loop. The performances were evaluated in the outer CV loop by computing the Area under the Receiver Operating Characteristic (ROC) curve (AUC) and accuracy. The metrics were computed within each fold; then, results across the test folds were used to calculate the mean and the standard deviation of accuracy and AUC.

### Explainability: identify important features

In order to identify the most significant features able to discriminate between ASD and TD, the explainable method SHAP [52], based on Shapley values computation, was adopted. SHAP is a local model-agnostic approach, since it uses only the input and the output of a classifier. The explanation of each feature is quantified in Shapley values ($\Phi$) and the importance of each feature in the DL model can be calculated by averaging the absolute values of the Shapley values for all instances as:

1 $$S = \frac{1}{N} \cdot \sum _{i=0}^{N} \Phi _i$$

where N is the number of instances in the dataset. We implemented the Gradient SHAP method, offered by the Python package SHAP^4^; this method uses the gradients of the model output with respect to input features to approximate Shapely values. Because we implemented a multimodal model using different inputs with different dimensionality, it was necessary to make comparable the SHAP values, by rescaling them in the same numerical range. Therefore, we performed a normalization with respect to the total sum of SHAP values and with respect to different number of input features (N$_{s,f}$). The normalized values were calculated as follows:

2 $$S_{norm} = \frac{S}{\sum _{i=0}^{N_{s,f}} S_i} \cdot \frac{N_{s,f}}{[2\cdot (N_{s} + N_{f})]}$$

The SHAP method was applied on the inner CV loop. To increase the robustness of results, the Shapely values were calculated using 100 different fold and the importance score were obtained as the average of the scores from 100 folds.

As the most important features, we selected the scores above the 99th percentile of importance features selected by SHAP. Moreover, the effect size of ASD vs. TD group difference was quantified using Cohen’s *d* coefficient. It consists in the standardized difference between two mean values $\mu$ defined as ($\mu _{\textrm{ASD}}$-$\mu _{\textrm{TD}}$)/SD$_{\text {pooled}}$, where SD$_{\text {pooled}}$ is the weighted average of the standard deviations of the two groups [19].

## Results

### Model performances

**Fig. 2:**
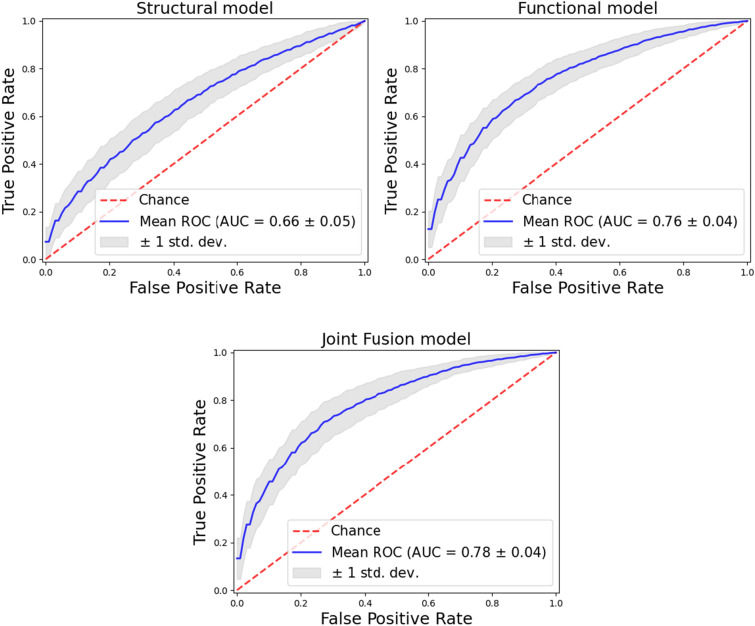
ROC curves obtained for the ASD vs. TD classification within 10-fold cross-validation scheme for the three different approaches: a structural DL model, a functional DL model and *joint fusion* DL model

The model was trained to distinguish subjects with ASD from TD according to a nested 10-fold cross-validation scheme. The classification performance was estimated both on the single data modality model (structural NN and functional NN) and on the multimodal *joint fusion* model in order to evaluate the improvement in discrimination capability. Figure 2 shows the ROC curves obtained by averaging the ROC curves computed on each of the 10 folds of the cross validation. The mean AUC values and the standard deviations are reported. The performance in the ASD vs. TD discrimination is reported in term of AUC and accuracy in Table 2.

**Table 2:** Classification performances obtained in the ASD vs. TD discrimination for the structural, functional and multi-modality model.

Type of model	AUC	Accuracy
Structural model	$0.66 \pm 0.05$	$0.75\pm 0.08$
Functional model	$0.76\pm 0.04$	$0.83\pm 0.12$
*Joint fusion* model	$0.78 \pm 0.04$	$0.85\pm 0.12$

The average value and the standard deviation of each metric are computed according to a nested 10-fold cross validation scheme

From the Table 2, it can be noticed an improvement of the performance using a multi-modality with *joint fusion* approach model, which outperforms the model based on functional features only ($p<0.001$). The superior performance is due to its ability to extract relationships among features from different modalities. Moreover, if we consider the single data modality, we can conclude that the functional model significantly outperforms the structural one, as known in the literature.

**Fig. 3:**
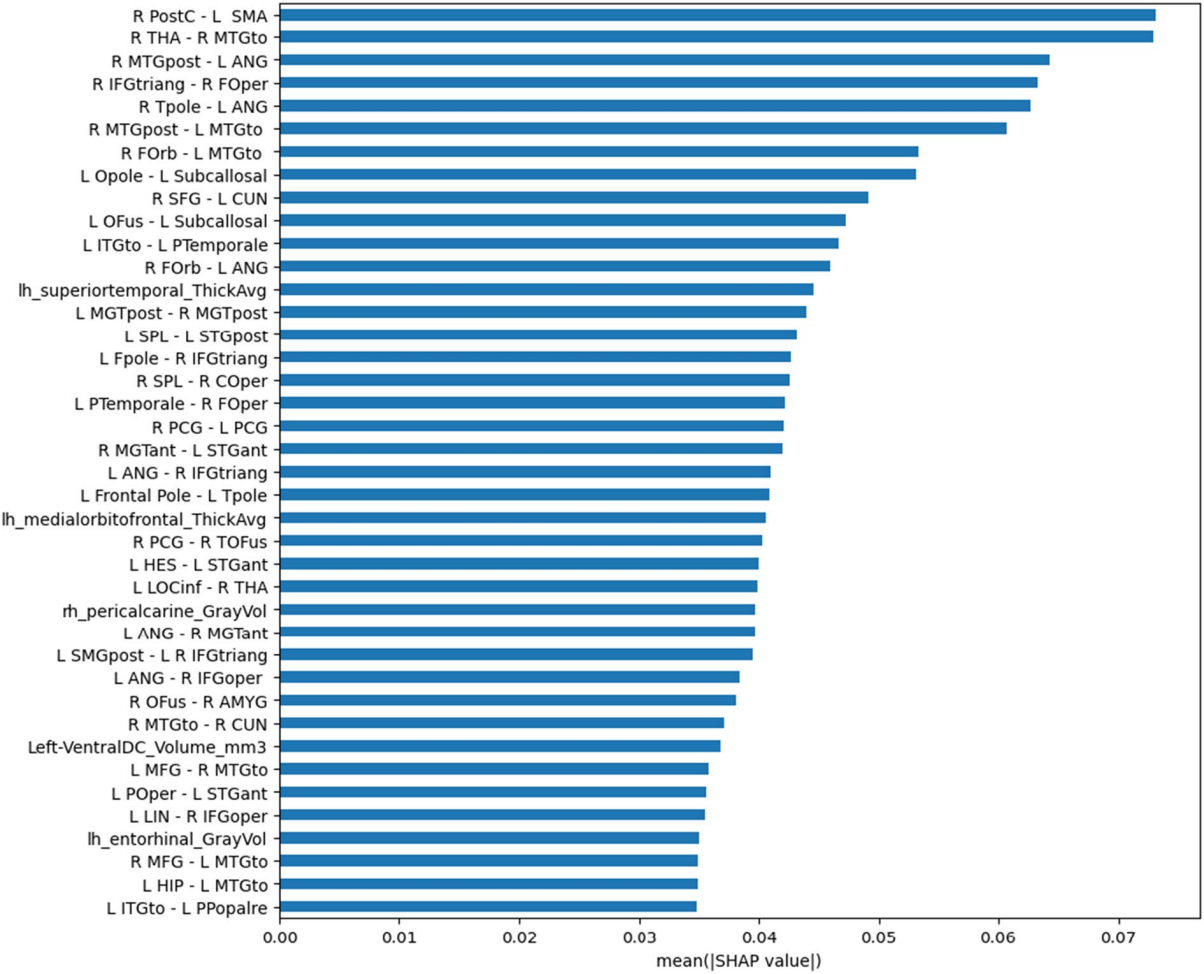
Boxplot of the top 40 importance features selected by SHAP. The functional labels are defined by the Harvard-Oxford cortical and subcortical atlases [48]

### Relevant brain features in the ASD vs. TD discrimination problem

**Fig. 4:**
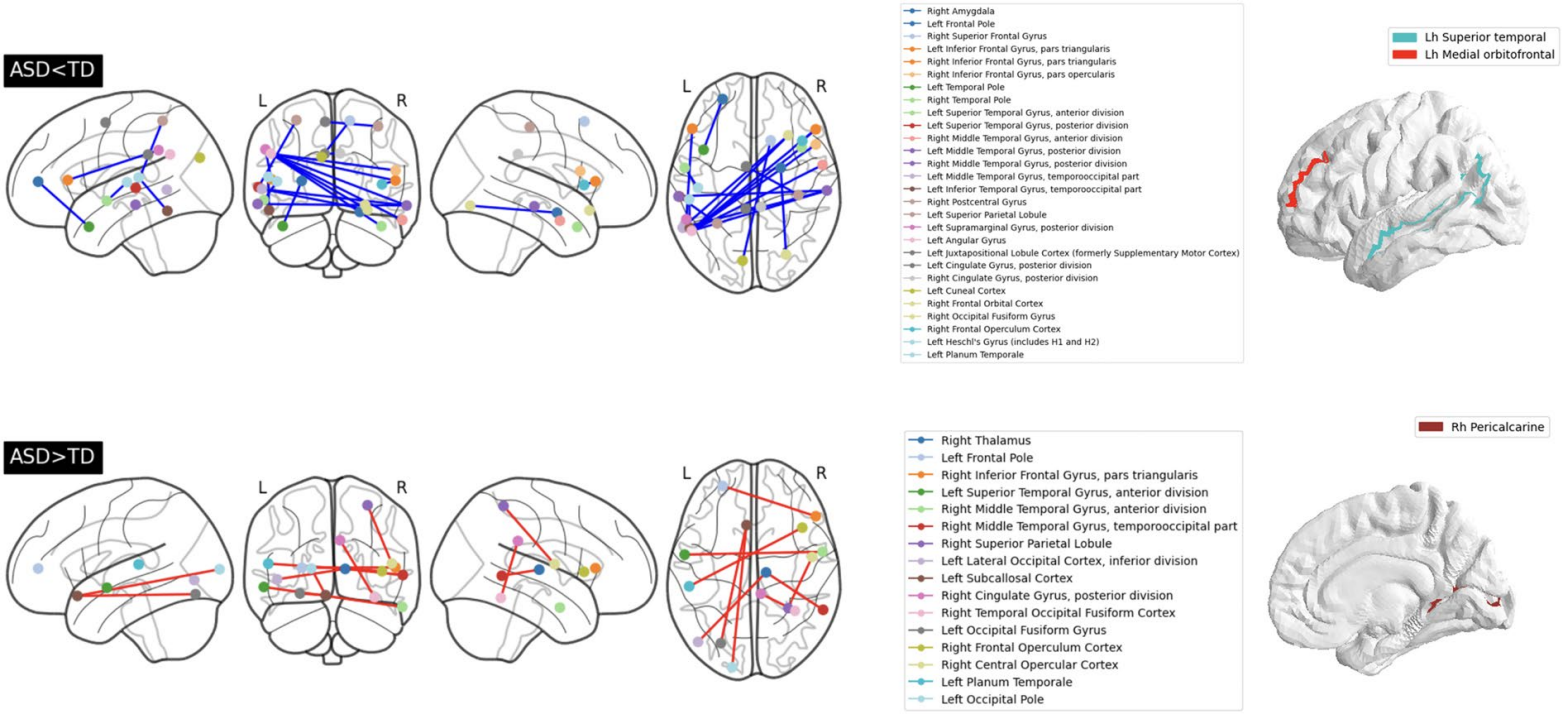
The most important features (see Table 3) in the ASD vs. TD discrimination are highlighted. Significant functional connections are reported in left box. The over-connectivity (in red) and under-connectivity (in blue) patterns are shown. In right box the brain regions whose features were identified as relevant are highlighted

The most important features in the ASD vs. TD discrimination problem were identified using Shapely values. The top 40 important features selected by SHAP are reported in Fig. 3. A list of the features whose importance scores exceeded the 99$^{th}$ percentile are reported in Table 3. In addition to the specification of the feature name, the table reports the sign of the Cohen’s *d*, thus indicating whether a feature mean is larger/smaller ($+/-$) in the sample of subjects with ASD with respect to TD controls. It can be noticed that the features identified as important in the ASD vs. TD discrimination problem were mainly from functional MRI data. A visual representation of the relevant features is shown in Fig. 4, which allows an immediate identification of the set of significant functional connections. We found out a long-range inter-hemispheric hypo-connectivity and an intra-hemispheric hyper-connectivity in ASD subjects with respect to TDs.

**Table 3:** The most important features (importance scores over the 99$^{th}$ percentile).

Brain Regions (Measurement)			Cohen’s d
Right Postcentral Gyrus	-	Left Juxtapositional Lobule Cortex	-
Right Thalamus	-	Right Middle Temporal Gyrus	+
Right Middle Temporal Gyrus (posterior division)	-	Left Angular Gyrus	-
Right Inferior Frontal Gyrus (pars triangularis)	-	Right Frontal Operculum Cortex	-
Right Temporal Pole	-	Left Angular Gyrus	-
Right Middle Temporal Gyrus (posterior division)	-	Left Middle Temporal Gyrus	-
Right Frontal Orbital Cortex	-	Left Middle Temporal Gyrus	-
Left Occipital Pole	-	Left Subcallosal Cortex	+
Right Superior Frontal Gyrus	-	Left Cuneal Cortex	-
Left Occipital Fusiform Gyrus	-	Left Subcallosal Cortex	+
Left Inferior Temporal Gyrus (temporooccipital part)	-	Left Planum Temporale	-
Right Frontal Orbital Cortex	-	Left Angular Gyrus	-
*Superior Temporal (ThickAvg)*			+
Left Middle Temporal Gyrus (posterior division)	-	Right Middle Temporal Gyrus (posterior division)	-
Left Superior Parietal Lobule	-	Left Superior Temporal Gyrus (posterior division)	-
Left Frontal Pole	-	Right Inferior Frontal Gyrus (pars triangularis)	+
Right Superior Parietal Lobule	-	Right Central Opercular Cortex	+
Left Planum Temporale	-	Right Frontal Operculum Cortex	+
Right Cingulate Gyrus (posterior division)	-	Left Cingulate Gyrus (posterior division)	-
Left Angular Gyrus	-	Right Inferior Frontal Gyrus (pars triangularis)	-
Right Middle Temporal Gyrus (anterior division)	-	Left Superior Temporal Gyrus (anterior division)	+
Left Frontal Pole	-	Left Temporal Pole	-
*Medial Orbitofrontal (ThickAvg)*			+
Left Heschl’s Gyrus (H1 and H2)	-	Left Superior Temporal Gyrus (anterior division)	-
Right Cingulate Gyrus (posterior division)	-	Right Temporal Occipital Fusiform Cortex	+
*Pericalcarine (GrayVol)*			-

The reported sign indicates whether the feature mean is larger/smaller ($+/-$) in the group of subjects with ASD with respect to TD controls. The structural features are highlighted in italics

## Discussion

We developed a multi-modality DL model with a *joint fusion* approach that uses the combination of structural and functional MRI data to discriminate subjects with ASD with respect to TDs. This DL model outperforms those based on each single modality. Several works demonstrated that better performance can be achieved by combining the results from structural and functional MRI data.

The work by Dekhil et al. [21] proposes a computer-aided diagnosis system that integrates features from sMRI and fMRI to predict autism diagnoses. They utilized traditional ML models, in particular, a local classifier (K-nearest neighbors) and a global classifier (Random Forest). The system was tested on 18 datasets from the ABIDE consortium, and the results demonstrated high accuracy ($0.75-1.00$ on sMRI and $0.79-1.00$ on fMRI data). However, the reported performances are specific to individual modalities and sites, lacking a comprehensive evaluation of the performance of the algorithm on combined data.

In their study, Aghdam et al. [5] utilized a deep belief network (DBN) to classify ASD subjects using both rs-fMRI and sMRI data. The study included participants aged 5 to 10 years from the ABIDE I and ABIDE II datasets. By combining rs-fMRI, gray matter, and white matter (WM) data, the study achieved an accuracy of 0.65, demonstrating that there were significant correlations between rs-fMRI and sMRI in diagnosing ASD. However, they employed an *early fusion* approach by combining the multi-modal features prior to the classification process. Despite a direct comparison between their work and ours cannot be done because of the different choice made in the selection of paticipants’ age range, we obtained substantially higher performances (an accuracy of $0.85\pm 0.12$) which may be attributable to the use of a *joint fusion* approach instead of an *early fusion* one.

Rakic et al. [67] propose a network consisting of autoencoders and multilayer perceptrons for the classification of ASD. The model was tested on both rs-fMRI and sMRI data from the ABIDE I dataset, both separately and in combination. They implemented both an *early* and a *late fusion* strategy, where connectivity and structural feature vectors were concatenated prior to classification (*early* approach) or classified separately and then the obtained label were fused (*late* approach). The best result was obtained using the *late fusion* approach, achieving a mean accuracy of 0.85, using an ensemble of 5 functional and 5 structural data classification models. This result is consistent with the performance obtained by our *joint fusion* model. However, late approach may potentially obscure valuable information that could be extracted from the interaction between modalities.

In their study, Abbas et al. [1] implemented a 3D model, which is completely different from ours and strongly more demanding from the computational point of view. The authors stated that they had implemented a subject partitioning criterion in the training, validation and test sets aimed at balancing the contribution of the different sites and diagnostic groups to each set. This approach to data partitioning may account for the fact that the ASD vs control classification performance they achieved is sensibly higher (AUC of 92.35) than ours.

Niu et al. [60] developed a multichannel deep attention neural network (DANN) model using functional neuroimaging data and personal characteristic data (e.g. sex, handedness, full-scale intelligence quotient) from ABIDE dataset. They achieved an accuracy of $0.73\pm 0.02$ in classifying subjects with ASD with respect to TDs. The results by Niu et al. [60] suggest that integrating additional data modalities can facilitate the utilization of ML in the context of computer-aided diagnosis of ASD. This work employed a *joint fusion* approach, however, the authors did not incorporate structural information. In our study, we obtained an improvement of the performance combining sMRI and rs-fMRI data.

To the best of our knowledge, our work is the first study to utilize a *joint fusion* approach on harmonized sMRI and rs-fMRI data, involving data from all 35 ABIDE sites. However, the diagnostic performance might be further improved by incorporating phenotype information. Additionally, we introduced the SHAP analysis within the framework of the multimodal model. This approach allows us to explore both the relative relevance and the interplay between structural and functional information in the ASD vs control DL discrimination task.

### Considerations of important features

Overall, our results revealed that individuals with ASD have poorer FC in brain regions spanning long, interhemispheric distances compared to TD controls, whereas FC seems to be increased in local, intrahemispheric circuits. This pattern has been firstly identified by Belmonte and colleagues [11] and confirmed by several subsequent independent investigations [8, 35, 41, 43, 56]. Among the most involved circuits, the weaker connection between pivotal hubs of the Default Mode Network (DMN) [66] including right middle temporal gyrus and left angular gyrus, right and left middle temporal gyrus, right and left posterior cingulate gyrus greatly contributed to distinguishing ASD participants from TD peers. Crucially, DMN is implicated in social cognition [55], theory of mind [17], emotional processing [15], self-evaluation [32], autobiographical memory ([59], and its disruption has been consistently described in subjects with ASD [9, 10, 24, 34, 79, 82]. Indeed, under-functional connectivity in regions of the DMN might contribute to the social-cognitive impairments associated with ASD. In addition, other brain regions that constitute the DMN, such as the superior frontal gyrus, the posterior cingulate gyrus, the superior temporal gyrus, the middle temporal gyrus, and the angular gyrus are part of the weaker connections we detected in individuals with ASD. Moreover, we observed atypical functional activation of areas belonging to the social brain, a network specialized in processing social cues and encoding human social behaviors [3, 14, 30], which includes the inferior frontal gyrus, the anterior cingulate cortex (subcallosal region), the superior temporal cortex, the temporal poles, and the fusiform gyrus. In line with our findings, previous investigations detected altered neural substrates in social brain [31, 62], which in turn may underlie abnormal processing of social cues, a hallmark of ASD.

It is also important to note a degree of overlap between the structural and functional findings of the current study: indeed, the left superior temporal gyrus (a crucial structure implicated in language and social cognition frequently impaired in ASD subjects [12, 13, 47]) is both increased in thickness and altered as far as FC is concerned in ASD individuals compared with control participants. This result support the notion that brain changes in ASD, even if subtle and diffuse, converge into specific, close localized areas of structural and functional alterations [58, 63, 69].

In our study, the classical case–control approach was implemented. However, it is crucial to acknowledge the limitations and challenges associated with this approach. Despite its widespread use, the classical approach did not take into account the intrinsic heterogeneity of ASD [50], presuming that the group mean is representative of the entire population. This assumption may not be valid for heterogeneous populations like ASD [54]. Recognizing the limitations of the classical approach, recent investigations tried to address the heterogeneity within ASD by exploring its biological underpinnings including neuroanatomical measures, with predominantly inconsistent results [4, 49, 85]. Moreover, a novel method for dealing with the neurobiological heterogeneity associated with ASD, and more broadly neurodevelopmental disorders, is normative modeling [70]. This method utilizes the trajectory of the typical developing brain across relevant variables to predict brain measures for each individual, highlighting deviations from the typical pattern for each individual. Normative modeling has been applied in previous investigations involving ASD individuals, revealing widespread patterns of deviations [73, 74, 84]. Given these considerations, it is evident that one of the most significant challenges in current ASD research is addressing and reducing the high heterogeneity at the neurobiological level in order to pave the way for more individualized treatment strategies for ASD individuals.

## Conclusions

In conclusion, our findings indicate that the DL-based *joint fusion* approach outperforms single modality DL models, as it can effectively exploit the complementary information encoded in each acquisition modality. The improvement in AUC demonstrated that sMRI and rs-fMRI images contain complementary information related to the ASD diagnosis. Furthermore, our work suggests that multi-modality DL models are promising tools for identifying potential neuroimaging biomarkers of neurodevelopmental disorders.

### Supplementary Information

**Additional file 1.** Sheet list of features: List of analyzed brain structural features. Sheet IDs of the participants: List of ID subjects per site selected in the final sample of the study.

## Data Availability

In this study the public dataset ABIDE was used. To allow the reproducibility of the analysis, the identification numbers (IDs) of the participants selected in the final sample are reported in Supplementary Materials.

## Abbreviations

ABIDE: Autism brain imaging data exchange
ASD: Autism spectrum disorders
AUC: Area under the curve
C-NN: Classification neural network
C-PAC: Configurable pipeline for the analysis of connectomes
DL: Deep learning
FR-NN: Feature reduction neural network
HO: Harvard-Oxford
IQR: Interquartile range
ML: Machine learning
MRI: Magnetic resonance imaging
ROC: Receiver operating characteristic
rs-fMRI: Resting-State fMRI
SDG: Stochastic gradient descent
SHAP: SHapley additive exPlanations
sMRI: Structural MRI
TD: Typical development

## Footnotes

1 https://surfer.nmr.mgh.harvard.edu/fswiki/recon-all

2 The extensive list of analyzed brain features can be found in the supplementary materials.

3 https://github.com/rpomponio/neuroHarmonize.

4 https://shap-lrjball.readthedocs.io/en/latest/generated/shap.GradientExplainer.html.
